# Effect of procalcitonin on the severity and prognostic value of elderly patients with a severe infection of oral and maxillofacial space

**DOI:** 10.1097/MD.0000000000030158

**Published:** 2022-08-26

**Authors:** Xin-yan Lin, Yu-zhao Lin, Shao-hua Lin, Jun-Jie Lian

**Affiliations:** a Department of Stomatology, Rongcheng Hospital Affiliated to Shandong First Medical University, Rongcheng, P.R. China; b Department of Infectious Disease, Rongcheng Hospital Affiliated to Shandong First Medical University, Rongcheng, P.R. China; c Respiratory and critical illness Department, Rongcheng Hospital Affiliated to Shandong First Medical University, Rongcheng, P.R. China.

**Keywords:** infection, oral and maxillofacial space, procalcitonin, prognosis

## Abstract

This study aimed to investigate the effect on the severity and prognostic value of serum procalcitonin for elderly patients with oral and maxillofacial infections. We divided 163 elderly patients with severe oral and maxillofacial infection into survival and death groups according to the prognosis between June 2015 and May 2021, measured serum procalcitonin by enzyme-linked immunosorbent assay on the 1st, 2nd, 3rd, 5^th^, and 7th day after admission for the dynamic changes of serum procalcitonin level, collected the general physiological and biochemical indexes for the scores of acute physiology and general chronic condition, compared the correlation between serum procalcitonin, mean platelet count and APACHE score, analyzed the prognostic value of serum procalcitonin levels at different time after admission by ROC curve. The serum procalcitonin level increased significantly in both groups after admission, sharply increased at first and then rapidly decreased in the survival group, and continued to rise or declined slowly with fluctuation of high level in the death group. There was a negative correlation between serum procalcitonin level and mean platelet count (r = −0.698, *P* < .05) and a positive correlation between serum procalcitonin and APACHE II (*R* = 0.803, *P* < .05). The ROC curve showed that the serum procalcitonin level had little value on the first day and great value on the third day in predicting the prognosis of elderly patients with severe oral and maxillofacial infection (PCT_1d_ = 0.539, PCT_3d_ = 0.875, *P* < .05). The serum procalcitonin level is correlated with the severity of the disease in elderly patients with severe oral and maxillofacial space infection. Dynamic observation of it is helpful for the prognosis judgment of patients. After admission, serum procalcitonin level on the third day has a great value for the prognosis judgment of elderly patients with severe oral and maxillofacial space infection.

## 1. Introduction

Oral and maxillofacial space infection, one of the common diseases of the oral and maxillofacial region, is an infection of an underlying fascial space, Most of which have some clinical manifestations such as skin redness, tissue swelling, elevated skin temperature, pain, limited mouth opening, and other local symptoms of maxillofacial soft tissue. The patient can recover quickly after active antibiotic treatment, local abscess incision, decompression, drainage, and other measures. A few patients become severe, resulting in bacterial sepsis as bacteria enter the blood circulation with toxins absorbed.^[1,2]^ The immune inflammatory reaction of elderly patients occurring sepsis can cause a series of distant organ and tissue damage, resulting in multiple organ failures and a high mortality rate.^[3]^ Recently, clinical data show that the incidence of sepsis caused by oral and maxillofacial space infection is on the rise, and clinicians have some problems such as “disorder of diagnosis and treatment, delay of opportunity” and so on.^[4,5]^ Some doctors have insufficient understanding of severe sepsis, inadequate initial treatment, and a lack of prediction of severity and prognosis in the process of treatment, which can easily lead to an increase in clinical mortality.^[6,7]^

The acute physiology and chronic health score are the objective indicators for the judgment of the severity of clinical conditions for the patients with critical illness, however, they are so complicated and inconvenient to be calculated and maneuverated.^[8]^ Clinically, there is an urgent need for a serum biological index that is simple to operate and easy to evaluate at the bedside to guide clinical treatment and judgment.

Serum procalcitonin is a precursor protein of calcitonin. Previous data show that its increase is highly related to bacterial infection.^[9–11]^ Its change can be used to guide the use of clinical antibiotics. It is not clear whether the serum procalcitonin level can reflect the severity of the disease and its change is beneficial to the prognosis of elderly patients with oral and maxillofacial infection, which is the purpose of the study.

## 2. Materials and Methods

### 2.1. Participants

A single-center retrospective study was carried out using a database that had been prospectively collected at the People’s Hospital of Rongcheng. One-hundred sixty three elderly patients (71 males, 92 females) with severe oral and maxillofacial infection from June 2015 to May 2021, age (61–89), mean age (68.95 ± 7.43). They were divided into survival group (127) and death group (36) based on patient prognosis according to their prognosis.

#### 2.1.1. Inclusion criteria.

I) clinical presence of infection local redness, swelling, heat, pain, and regional lymphadenopathy,
II) The elevation of the total number of leukocytes in blood routine, left shift of its nucleus under the microscope, CRP >40 mg/L.

#### 2.1.2. Exclusion criteria.

I) initial onset of acute systemic infection at other sites such as acute pneumonia, urinary infection, etc
II) patients with immune connective tissue diseases.

Ethics: This study was approved by the ethics committee of Rongcheng people’s Hospital of Shandong Province, people’s Republic of China (Clinical Research Registration Approval No. 2019-13). All patients who would like to take part in this study signed a consent form. We guaranteed the right of patients or their families to know about the disease and obtained their written consent.

### 2.2. Definitions

All patients were diagnosed with severe infection of oral and maxillofacial space by at least 2 consultant infection specialists independently, with the help of clinical syndrome, laboratory tests (the presence of WBCS and procalcitonin), ultrasonic examination results or radiological evidence, and microbiological documentation. The diagnosis of severe infection was consistent with the International Sepsis Forum Consensus Conference standard. It is defined as SIRS plus the evidence of focal infection. Diagnostic criteria for SIRS : temperature >38°C or <36°C, heart rete > 90 beats/min, respiratory rate > 20 breaths/min or PaCO2 < 32 mm Hg, and WBC count >12,000/mm^3^, <4000/mm^3^. Patients were diagnosed with SIRS when presenting at least 2 or more of the following conditions.

### 2.3. Detection methods and data collection

After enrollment, all the demographic data, which included age, sex, diabetes (yes or no), hypertension (yes or no), chronic coronary heart disease (yes or no), body mass index, and so on, were recorded respectively. The general physiological and biochemical indexes were collected to calculate the scores of acute physiology and general health status of patients. The serum procalcitonin level was detected and measured by an enzyme-linked immunosorbent assay (LUMtest PCT; Brahms Dianostica, Berlin, Germany) on the 1st, 2nd, 3rd, 5^th^, and 7th day after admission, and the dynamic changing trend of serum procalcitonin level was observed. Average platelet counts were measured via the Hematology Analyzer (XS-1000, Sysmex, Kobe, Japan). All samples were processed and analyzed on the spot.

### 2.4. Statistical Analysis

Data were analyzed by SPSS21.0 statistical software, and the measurement data were expressed by ($\bar{x}\pm s$). Continuous variables were compared using the 2-sample *t*-test between the 2 groups. Pearson correlation coefficient was used to compare the correlation among serum procalcitonin, average platelet count, and APACHE II score. Receiver operating characteristic (ROC) curve and area under the curve (AUC) was performed to assess the predictive power of serum procalcitonin levels at different time points after admission in elderly patients with severe oral and maxillofacial infection. *P*-values < 0.05 (two-sided) indicated that the difference is statistically significant.

## 3. Results

### 3.1. Comparison of serum procalcitonin levels between patients in 2 groups

**Figure 1.**
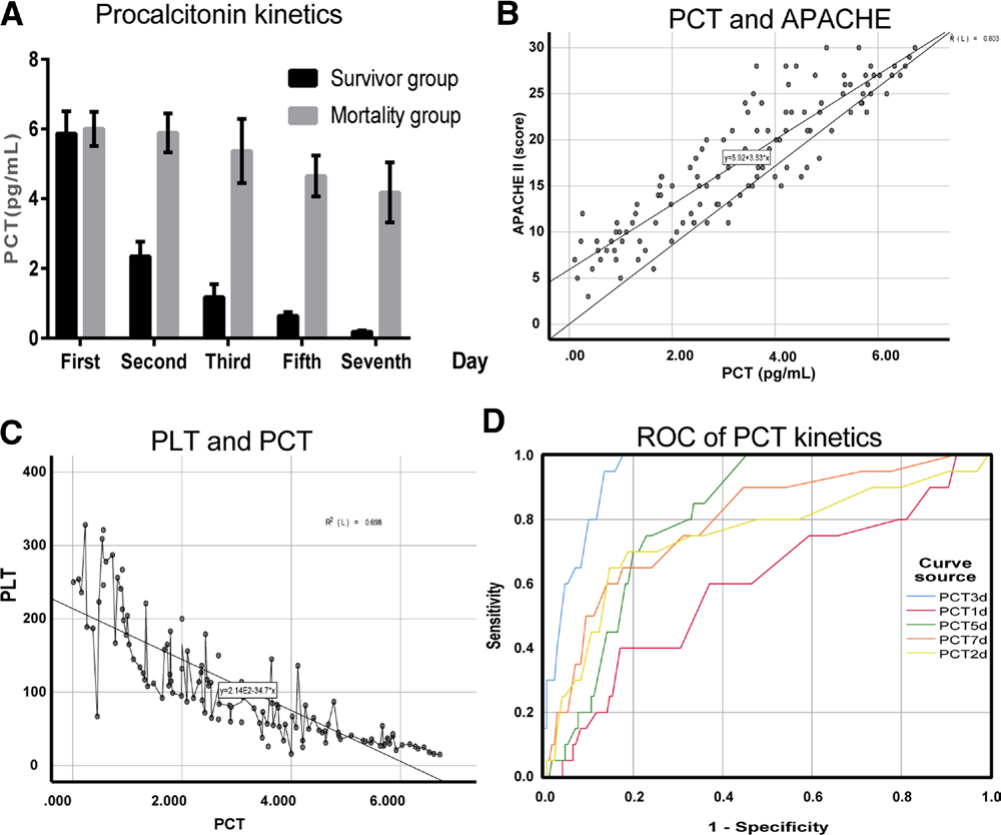
A: Comparison of serum procalcitonin levels between patients in 2 groups: After admission, the serum procalcitonin level was significantly increased in 2 groups, while increased sharply at first and then decreased rapidly in the survival group and, fluctuated continuously, or increased continuously, or decreased slowly in high conditions in the death group. B: The serum procalcitonin level was correlated positively with APACHE II *(R* = 0.803, *P* < .05). C: The serum procalcitonin level was correlated negatively with the mean platelet count (*r* = - 0.698, *P* < .05). D: PCT predictive values at different time points: ROC curve showed that the level of serum procalcitonin had little value on the 1st day and great value on the 3rd day in predicting the prognosis of elderly patients with severe oral and maxillofacial infection (PCT1d = 0.539, PCT3d = 0.875, *P* < .05).

After admission, the serum procalcitonin level was significantly increased in 2 groups, while increased sharply at first and then decreased rapidly in the survival group and, fluctuated continuously, or increased continuously, or decreased slowly in high conditions in the death group, as shown in Table 1, Figure 1A.

**Table 1:** Comparison of serum procalcitonin level between patients in 2 group.

Variable	n	PCT (pg/mL)				
		1st	2nd	3rd	5th	7th
SG	127	5.87 ± 0.64	2.35 ± 0.42	1.18 ± 0.37	0.64 ± 0.11	0.19 ± 0.03
DG	36	6.01 ± 0.49	5.89 ± 0.56	5.37 ± 0.92	4.65 ± 0.59	4.18 ± 0.86
*t*	–	1.214	41.28	41.13	72.78	52.59
*p*	–	>0.05	<0.000	<0.000	<0.000	<0.000

DG = Death group, PCT = serum procalcitonin, SG = survival group.

### 3.2. Correlation Analysis

The serum procalcitonin level was correlated negatively with the mean platelet count (*r* = - 0.698, *P* < .05) and positively with APACHE II *(R* = 0.803, *P* < .05) (shown in Fig. 1B, C).

### 3.3. Prognosis Judgment

ROC curve showed that the level of serum procalcitonin had little value on the 1st day and great value on the 3rd day in predicting the prognosis of elderly patients with severe oral and maxillofacial infection (PCT_1d_ = 0.539, PCT_3d_ = 0.875, *P* < .05) (shown in Fig. 1D).

## 4. Discussion

The infections of oral and maxillofacial space are prone to occur in a population of any age, most are confined in the soft tissue or underlying fascial space of the maxillofacial region mainly with local symptoms. Few patients are severe which can cause a systemic systemic inflammatory response, sepsis, severe sepsis, septic shock, and systemic symptoms, especially in the elderly patients. Sepsis is the main cause of morbidity and mortality worldwide.^[12]^ Data analyzed that more than 4% of the hospitalized patients have been diagnosed with sepsis. There was a marked difference in the range of sepsis in different age groups. Elderly patients have many serious basic diseases such as hyperglycemia, hypertension, coronary heart disease, the organ functional reserve decreased, and so on. At the same time, improper treatment of pericoronitis of wisdom teeth or irregular extraction of third molars is common cause of infection in the maxillofacial subfascial space which can easily lead to sepsis.^[13,14]^ Sepsis, with its high mortality and high disability rate, mainly caused by bacterial infection, is one of the leading causes of mortality in patients in modern intensive care units.^[15]^ Bacterial infection is the leading cause of sepsis. After bacteria or toxins enter the blood circulation, they cause immune-inflammatory mechanism cascading waterfall-like outbreaks, which release a large number of cytokines and inflammatory mediators, resulting in excessive systemic inflammatory response syndrome.^[16]^

Patients with severe sepsis are prone to distant organ insufficiency and inadequate blood perfusion to the visceral tissues because of immune-inflammatory injury, with an extremely high incidence of multiple organ function injury or failure, they need comprehensive and cluster treatment during the initial stage.^[17]^ The timely identification of the severity of the disease and the accurate judgment of the prognosis is beneficial to the timely adjustment and improvement of the treatment plan. Studies have shown that most of doctors believe that the clinical manifestations of sepsis are not typical.^[18]^ The lack of suggestive clues of specific symptoms and signs that attract the attention of clinicians leads to the delay of clinical diagnosis and treatment. Improving the ability of early clinical recognition, accurately evaluating the severity of the disease, and implementing reasonable treatment strategies as soon as possible is the key to preventing and curing the deterioration of sepsis and improving the prognosis.

Because sepsis is a clinical syndrome with higher mortality demonstrating a remarkable relationship to illness severity, the further study of noteworthy and reliable biomarkers of sepsis has been the focus field of interest.^[19]^ Blood culture is the gold standard for the diagnosis of bacteremia and sepsis. However, it is time-consuming, has low sensitivity, and can not achieve the purpose of rapid diagnosis, which limits its clinical application. The search for clinical practical and reliable biomarkers has become a hot spot in clinical research. The most significant feature of a sound biomarker has its potential influence on clinical decision-making. Procalcitonin, interleukin-6, C-reactive protein, and other biological markers have been the favorites of research pursuits.^[20]^ Procalcitonin is one such marker that has shown great promise in identifying sepsis, assessing the prognosis and severity, and guiding appropriate treatment of the illness.^[21]^ Procalcitonin is a small molecular polypeptide and a part of the inflammatory cascade in all kinds of sepsis, which is mainly secreted by thyroid C cells under normal circumstances. It is reported that the level of procalcitonin showed an obvious regular pattern of change. Under normal circumstances, procalcitonin can be easily detected within 4 hours and has a half-life span of 20-26 hous.^[22]^ However, it is also synthesized and secreted by systemic tissue cells in microbial infection and other inflammatory reactions such as surgery, tumor and trauma, and so on. The unique biological characteristics make the procalcitonin response to body infection fast and accurate. Some studies believe that the level of serum procalcitonin has an important role in the early diagnosis and antibiotic treatment of sepsis, but there is still a lack of research on its impact on the recognition of disease severity and prognosis for elderly patients with oral and maxillofacial infection.^[23]^ 163 elderly patients with severe maxillofacial infection were retrospectively analyzed and divided into 2 groups according to their prognosis. The level of serum procalcitonin increased significantly in both groups after admission. The result shows that evaluated procalcitonin as a marker of infection with oral and maxillofacial infection also has significantly higher specificity. Because the main pathogens of oral and maxillofacial infection are gram-positive aerobes, the proportion of gram-negative aerobes is low. Previous studies have reported the increase in procalcitonin level is closely related to Gram-negative bacilli. PCT levels were higher in patients infected with gram-negative bacteria than in patients with gram-positive aerobes. Therefore, it can be used as a clinical index for patients with severe maxillofacial infection complicated with sepsis, although its clinical value has always been doubtful.

There have been some conflicting data about the utility of procalcitonin as a predictor of patient prognosis in clinical.^[24,25]^ Some studies demonstrated a close association between evaluated procalcitonin and the severity of illness. Other studies also showed that the lack of a significant relationship between initial PCT level and patient outcomes is disappointing. The evolution of PCT as a biomarker of the patient with severe maxillofacial infection is still unclear. The current literature suggested a promising revelation that procalcitonin kinetic is a more efficient marker of prognosis than static data or one-time measurements. Continuous dynamic monitoring showed that the serum procalcitonin level increased sharply, then decreased rapidly in the survival group and fluctuated continuously, either increasing continuously or decreasing slowly in the death group. Platelet count and APACHE II score are recognized as effective evaluation indexes for patients with severe infection. The lower the platelet count, the higher the APACHE II score and the more severe the infection. The results showed that it, with the evolution of the patient’s condition, had a correlation that was negative with the average platelet count and positive APACHE II. The average platelet count of patients with severe infection lowed significantly due to immunosuppressive injury, its decrease was a linear negative correlation with the severity of infection, that is, the heavier the infection, the lower the number of platelets. The results showed that the level of serum procalcitonin was significantly correlated with the severity of infection, its rapid decrease and fast increase of average platelet level indicated that the infection was effectively controlled, which were the important indicators for the improvement of patients’ condition. Therefore, dynamic monitoring of procalcitonin level is also beneficial to judge the severity of the disease, adjust the treatment plan in time and reverse the prognosis of patients.

PCT kinetics were acknowledged to be of prognostic value for the patient with sepsis. However, although an initial or static PCT level may be useful in the determination of illness severity, it may not always be clear whether the PCT value can be a reliable prognostic and standalone value. In particular, monitoring involves different time points.^[26]^ Further analysis of the ROC curve showed the level of serum procalcitonin had little value on the first day and great value on the 3rd day in predicting the prognosis of elderly patients with severe oral and maxillofacial infection. The results showed that although it only reflected the severity of infection at the beginning of admission and had little relationship with the prognosis of the patients, its dynamic monitoring will help to improve the survival rate and improve the prognosis of patients during treatment and timely adjustment of a treatment plan. The guidelines for the treatment of sepsis emphasize the importance of early comprehensive and cluster treatment.^[27]^ The timing of treatment is particularly important for judging the prognosis of the disease, the key to the prevention and treatment of secondary organ persistent injury within 6-hour in the early stage of infection, and the basis for a good prognosis within 72-hour. The changing trend of serum procalcitonin on the third day after admission may become a watershed to judge the outcome of the disease, which is also in line with this guiding principle. It was reported that a statistically significant difference was noted with regard to antibiotic use under the guidance of procalcitonin and traditional empiric antibiotic therapy. Future studies on the current topic are also therefore recommended.

To sum up, the present results are significant in at least 2 major respects. First of all, the level of serum procalcitonin is related to the severity and is of great value in predicting the prognosis of elderly patients with severe oral and maxillofacial space infection. Secondly, Dynamic observation of its changing trend is helpful to judge the prognosis of patients.

## Acknowledgments

The authors thank the patients who participated in this study and the staff involved in this work. This work was also supported by the Rongcheng Hospital, affiliated with the Shandong First Medical University.

Funding: There was no funding granted for this article.

Competing interests: None.

## Author contributions

Conceptualization: Xin-yan Lin

Investigation:Yu-zhao Lin, Shao-hua Lin.

Methodology: Xin-yan Lin

Software: Shao-hua Lin.

Writing – original draft: Xin-yan Lin

Writing – review & editing: Jun-Jie Lian

All authors read and approved the final version
